# Supplementary material for: Does Artificial Intelligence Make Clinical Decision Better? A Review of Artificial Intelligence and Machine Learning in Acute Kidney Injury Prediction
Source: Healthcare (Basel). 2021 Nov 30;9(12):1662. doi: 10.3390/healthcare9121662 (PMC8701097; doi:10.3390/healthcare9121662)
Supplement: Supplementary file 1 [file healthcare-09-01662-s001.zip › healthcare-1448535-supplementary.pdf]

Supplementary Table S1. Details of Search Strategy Source: Pubmed; Searched on: 01, Dec, 2020;

Search	Query	Items
#1	"acute kidney injury"[Mesh]	25357
#2	"acute kidney injury"[Title/Abstract]	37559
#3	"machine learning"[Title/Abstract]	39514
#4	“machine learning” [Mesh]	24175
#5	“artificial intelligence” [Title/Abstract]	11145
#6	“artificial intelligence” [Mesh]	107310
#7	#1 OR #2	59155
#8	#3 OR #4	49985
#9	#5 OR #6	113598
#10	#8 OR #9	133529
#11	#7 AND #10	145
